# Barriers to optimal management of cancer pain in sub-Saharan Africa: a scoping review

**DOI:** 10.3332/ecancer.2023.1650

**Published:** 2023-12-20

**Authors:** Vivian Onyinyechukwu Magboh, Ogheneochuko Andrew Saba, Rene Krause, Patrice Forget

**Affiliations:** 1 School of Medicine, Medical Sciences and Nutrition, Institute of Applied Health Sciences, University of Aberdeen, AB24 3FX Aberdeen, UK; 2 26 Charlotte Street, AB25 1LQ Aberdeen, UK; 3 Division of Interdisciplinary Palliative Care and Medicine, Department of Family, Community and Emergency Care, Faculty of Health Sciences, University of Cape Town, Observatory 7925, South Africa; 4 Epidemiology Group, School of Medicine, Medical Sciences and Nutrition, Institute of Applied Health Sciences, University of Aberdeen, AB25 2ZD Aberdeen, UK

**Keywords:** cancer, pain, barriers, cancer pain management, sub-Saharan Africa

## Abstract

**Background:**

Although cancer is a significant issue in sub-Saharan Africa, and cancer pain is prevalent, there is insufficient data and research on the barriers to cancer pain management. Even in countries where evidence exists, few studies explore the links between these barriers, which makes it difficult to implement system-wide approaches to address them.

**Methods:**

The search strategy was developed and conducted on databases including MEDLINE, Embase and Web of Science to identify peer-reviewed studies. Then, these retrieved studies were screened systematically to select papers that had met pre-specified criteria. The barriers were categorised into patient-, health professional- and health system-level domains. Then, the quality of the included papers was assessed using the mixed methods appraisal tool. Finally, a narrative synthesis was utilised to summarise the findings.

**Results:**

Fourteen relevant articles from 19 sub-Saharan African countries were included in the scoping review. All the studies highlighted barriers to optimal cancer pain management. Healthcare system-related domains had the most frequently reported barriers. Approximately half of the included studies met 100% of the methodological quality criteria in the critical appraisal.

**Conclusion:**

Improving pain management for cancer patients in sub-Saharan Africa requires further high-level research evidence on regulatory policies and interventional strategies, especially at the health system level, as most barriers to cancer pain treatment essentially stem from the healthcare system.

## Background

According to the World Health Organisation (WHO), cancers are one of the leading causes of morbidity and mortality globally, with approximately 18.1 million new cases and 9.6 million deaths in 2018 [1]. In sub-Saharan Africa, cancer is a significant public health problem affecting about 1 billion of its population, with an estimated 801,392 new cases and 520,158 deaths as of 2020 [2].

*Pain* is ‘an unpleasant sensory and emotional experience associated or resembling that associated with actual or potential tissue damage’ [3]. It is multidimensional and complex, involving physical, psychological, spiritual and emotional factors [4]. Cancer-related pain is described as pain associated with the cancer itself, treatment modality or adverse effects of the treatment [5, 6]. It is now a well-described entity, recognised by the WHO in the 11th International Classification of Disease (ICD-11) [7]. Cancer pain is one of the most prevalent and debilitating symptoms and is estimated to be the first presenting complaint in about 30% of cancer patients [8, 9].

The WHO guiding principles for cancer pain management state that the goal of optimum pain management is reducing pain to levels that permit an acceptable quality of life [1]. Alternative interventions for improving the adequacy of cancer pain relief exist; however, pharmacological therapy remains the mainstay of treating cancer pain [1]. In 1984, the WHO analgesic ladder was developed to guide adequate cancer pain management. This ladder advocated for initiating pain medication stepwise and was created based on the fundamental principle for pain management – ‘By mouth, by clock, by individual’ [10]. However, not all research agrees with a simplified approach due to the multicomplexes of cancer pain. Some evidence argues that pain assessment and severity score should generally be considered when prescribing analgesics to ensure optimal pain control in cancer patients. Thus, the analgesic ladder has recently been re-debated, with the alternative being a more comprehensive strategy [1, 11].

Despite these scientific debates, barriers to cancer pain relief remain a problem worldwide, especially in resource-limited regions due to cultural, political and socio-economic complexities associated with health outcomes and service delivery [5, 12]. Sub-Saharan Africa faces unique challenges in cancer pain management due to various multifaceted factors such as limited funding for palliative care services, inadequate healthcare infrastructure and lack of trained healthcare professionals [13]. Furthermore, inadequate accessibility and availability of opioids remain a significant problem in this region because of strict regulatory measures [13, 14]. Thus, potential solutions would require a critical analysis of the already existing linkages between these barriers reported in literature and system-level thinking to address them holistically.

There is no systematic or scoping review of the barriers to optimal cancer pain management in sub-Saharan Africa. Hence, this scoping review aims to provide an overview of reported barriers to cancer pain management in sub-Saharan Africa and linkages between these barriers. It will also highlight areas where further research is needed, allowing policymakers, healthcare providers and researchers to focus their efforts and resources on addressing those gaps. This review sought to answer three key research questions:

1. What is the existing evidence on sub-Saharan Africa's reported barriers to cancer pain management?
2. What connections between these barriers exist in literature?
3. What are the opportunities for intervention and further research to optimise cancer pain management in sub-Saharan Africa?

## Methods

This scoping review was conducted between May 2023 and July 2023. The reporting of the scoping review followed the Preferred Reporting Items for Systematic Reviews and Meta-analysis Extension for Scoping Reviews guidelines [15]. While the guidelines encourage a ‘review registration’ step and writing of a protocol, these were not done since scoping reviews cannot be registered in the PROSPERO database. Additionally, summary measures, risk of bias and additional analyses sections were not included because these are not applicable to scoping reviews.

### Eligibility criteria

For an article to be eligible for review, it had to:

1. Discuss existing evidence on barriers to the optimal management of cancer pain in sub-Saharan Africa.
2. Discuss barriers to opioid availability and accessibility for cancer pain in sub-Saharan Africa.
3. Be a peer-reviewed primary study.
4. Be conducted in an adult population.
5. Be published in English.
6. Be published from January 2000 to 4th of July 2023.

### Information sources

To conduct a literature search for systematic reviews, some researchers recommend the use of at least three database combinations [16]. Accordingly, three electronic databases containing millions of references from health-related journals namely Embase, MEDLINE and Web of Science (WoS) were searched to identify relevant articles.

### Search strategy

**Figure 1.**
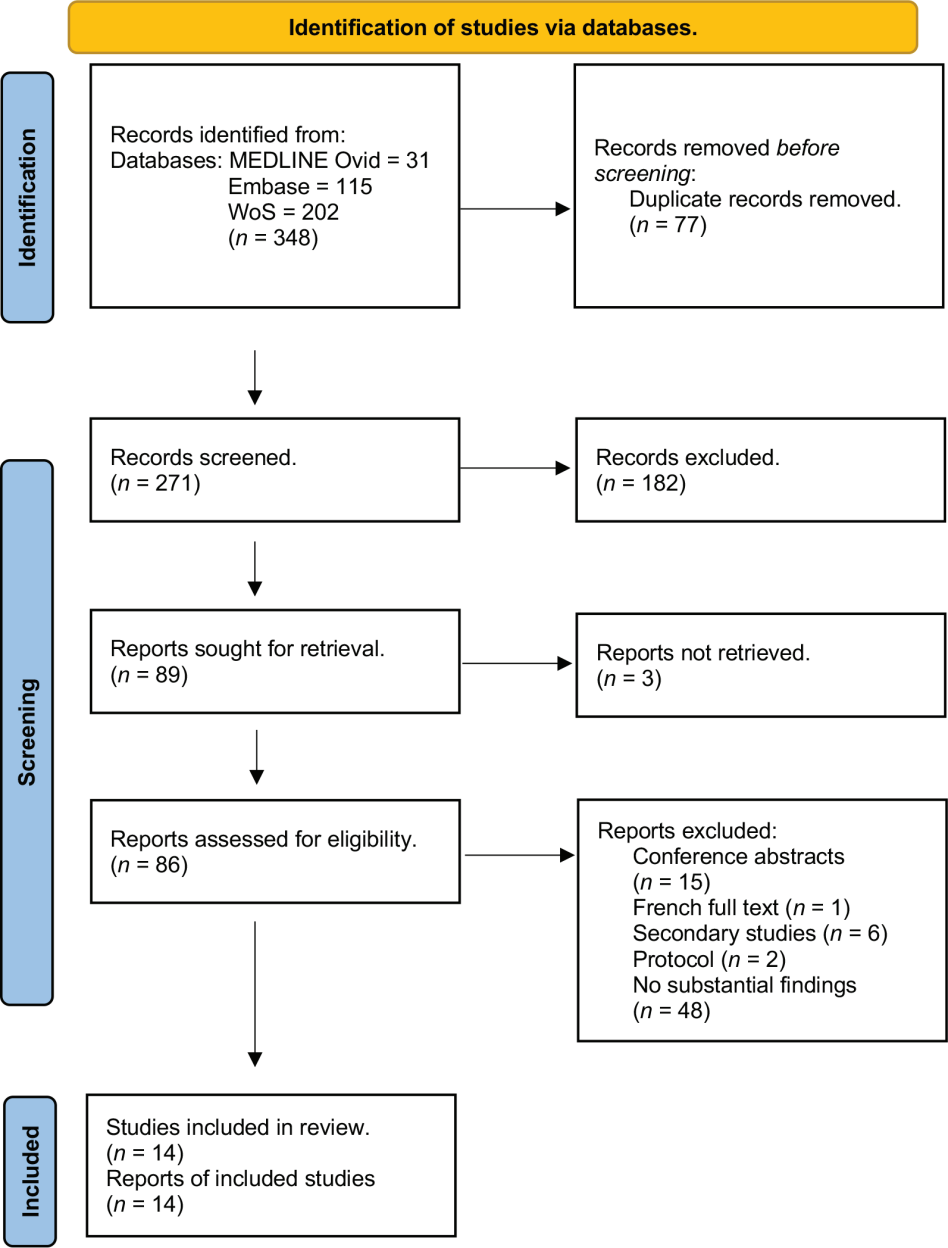
Flow diagram (adapted from [18]).

A comprehensive online database search was done on MEDLINE, Embase and WoS to identify peer-reviewed primary studies. These searches were conducted in July of 2023. Keywords such as cancer pain, barriers, pain management, pain control, pain relief, Africa and south of Sahara Africa were used. This strategy was applied uniformly across all databases. The full search strategy can be viewed in Appendix 1. The complete search and screening process is depicted in Figure 1.

### Selection of sources of evidence

After a final search on each database, 348 articles were exported to RefWorks, 77 duplicate reports were eliminated and the titles and abstracts were assessed for eligibility. Two researchers independently did a two-staged screening, and the project supervisor resolved any disputes. The first stage involved the independent screening of titles and abstracts. A consensus meeting was held afterwards, and the project supervisor resolved disagreements that could not be reconciled after discussion. The second stage involved the screening of full texts of studies that were included in the first stage. The screening was done in the same format as described in the former.

### Data charting

Following the screening, a comprehensive and rigorous analysis of the eligible research papers was conducted, and general study characteristics, such as author names, publication dates, study design, study population and geographic location of the studies, were extracted. All the relevant data were organised systematically in a Microsoft Excel spreadsheet. The key variables such as the reported barriers explored in the articles were carefully analysed, and the overarching domains identified from the papers were patient-related, health care professional-related and health care system-related. A subset of these outcomes discussed in the eligible papers was selected to ensure relevance and brevity.

### Critical appraisal of individual sources of evidence

To assess the quality of the studies, the mixed methods appraisal tool (MMAT) version 2018 created by Hong *et al* [17] was utilised. This was done independently by two researchers. A consensus meeting was held afterwards and disagreements that could not be reconciled after discussion were resolved by the project supervisor. The MMAT was developed and validated for the standardisation of the quality assessment for systematic mixed studies review such as this scoping review. The tool has a subset of five questions for each study design which are rated as ‘Yes’, ‘No’ or ‘Can’t tell’. The responses were coded as 0 (No and Can’t tell) or 1 (Yes). Then, according to the reporting suggestions for the MMAT v.2018 tool, a star rating was devised to report the percentage of the quality criteria achieved by a retained study. This was done because calculating an overall score for each study is not informative. Five stars meant that 100% of the quality criteria were met, four stars meant 80%, three meant 60%, two meant 40% and one meant 20%. For mixed method studies, there are 15 criteria to score instead of five. Thus, it is advised that the overall quality score for a mixed method study is equal to that of the weakest component.

### Synthesis of results

The systematic database search and screening process results were summarised in the flow diagram [18]. Tables were used to summarise the general characteristics of included studies and the essential findings and quality ratings of each study separately. A narrative synthesis was used to analyse the results. The barriers were categorised in domains based on the 1994 Agency for Healthcare and Policy Research classification for barriers to optimum pain relief [19]. Additional features that were not included in these tables were described in a narrative format.

## Results

### Selection and characteristics of sources of evidence

**Figure 2.**
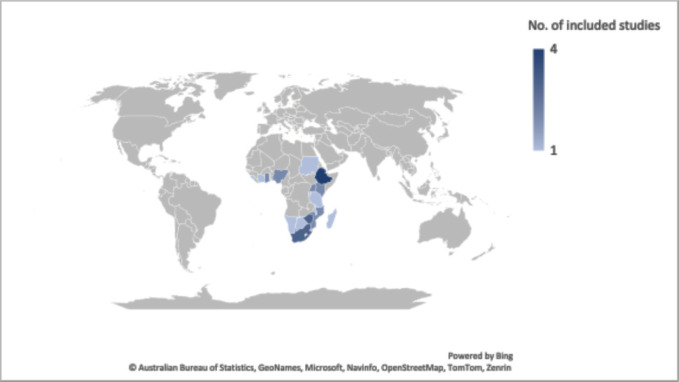
Sub-Saharan African countries with reported barriers to cancer pain management.

Overall, 14 studies met the inclusion criteria and were included in this review as shown in the flow diagram (Figure 1). The geographical distribution of the included studies is illustrated in Figure 2.

Most of the included papers were cross-sectional studies (10/14) and conducted in multiple settings. They utilised methods like questionnaires, in-depth interviews, key informant interviews, surveys and document reviews. The main characteristics of the included studies are illustrated in Table 1. Cancer patients were the commonest population studied although, some studies were conducted on other stakeholders of health such as health service providers and governmental representatives. Furthermore, two were multi-country studies [20, 21] while the remaining 12 were conducted in individual countries within sub-Saharan Africa [22, 23] as depicted in Table 1.

### Critical appraisal of sources of evidence

Overall, more than half of the included studies (*n* = 8) satisfied 100% of the methodological quality criteria set in the quality assessment as highlighted in Table 2.

### Synthesis of results of individual sources of evidence

All the studies investigated barriers to optimal cancer pain management. Although, different factors were classified across the three domains of pain management barriers, at least two domains were significantly represented in more than half of the eligible papers. Healthcare system related barriers were the most frequently reported barriers in the included studies as denoted in Table 2.

## Discussion

### Summary of evidence

There is a fair geographical distribution of the included studies with 19 out of 46 sub-Saharan African countries represented in at least one study. Ethiopia, Uganda, Kenya and South Africa had the highest representation in this review. This is thought to be because evidence suggests that South Africa is one of the three countries reported to have the highest burden of cancer incidence and deaths [2]. It is also the most research advanced country in the sub-region with more scientific publications and experience with grant application for research alongside, Ethiopia, Uganda and Kenya which also feature in the top ten countries with the highest number of studies and peer-reviewed articles in reputable journals [34]. On the other hand, the lack of findings from the other countries not represented in this review is possibly due to limited funding and low political will for research, ongoing conflicts such as in Somalia and Central Africa Republic, and the restrictive language criterion that excludes publications from Francophone countries in the sub-region [34].

Findings from this review may further suggest that the current literature about cancer pain management in sub-Saharan Africa is focused on identifying the problems, describing issues and stating opinions rather than conducting rigorous analytical research.

The reported barriers analysed in this review were categorised into three domains to enable further discussion and conceptualisation of the linkages between the key findings.

### Patient-related barriers

Cultural beliefs and practices and out-of-pocket (OOP) expenditure were the commonest barriers reported by cancer patients. In many African settings, culture influences the interpretation and understanding of pain [35]. For instance, a South African study revealed that cancer patients often believe that the process of dying is expected to be inevitably painful and should be endured while a Ghanian study highlighted that patients believed cancer pain should be tolerated [24, 33]. Also, patient’s cultural perspectives have been shown to influence the use of traditional medicines or non-pharmacological therapies like spiritual beliefs [36]. Interestingly, traditional medicines were reported to further deteriorate patients’ clinical condition and worsened their pain whereas, spiritual beliefs were found to have a positive impact on cancer pain reduction [22, 37]. Findings from this study are similar to results from a systematic review which highlights the normalisation of cancer pain among patients with African descent due to their shared cultural beliefs [36].

Regarding OOP expenditure, the healthcare system in many sub-Saharan African countries lack financial resources for universal health coverage [24]. Hence, only those who can afford to pay for the cost for healthcare services could have access to pain medications [30]. This finding is also corroborated by another review where cost was highlighted as the major contributory factor to delays in cancer care pathway in sub-Saharan Africa [38].

### Health professional-related barriers

Health professionals face many challenges to optimal cancer pain management in sub-Saharan Africa. Opiophobia also known as ‘fear of opioids’ was frequently discussed across the studies included in this review. Physicians fear that opioids may interfere with pain assessment, patients may develop tolerance and cancer pain interventions will conceal the advance in cancer treatment [28]. Likewise, health workers’ fears of possible addiction, overdosing and serious side effects related to opioid administration to patients often result in the undertreatment of cancer pain [24, 27]. It is also important to emphasise the interconnection between the barriers faced by health professionals. The limited knowledge and negative attitudes to cancer pain management among health professionals in some countries within the region has been attributed to the fact that health workers had received no formal training on pain management [21, 27, 28]. Thus, even when medications are available, inadequacies in pain assessments and opioid prescription were common among service providers [31]. Similar findings in literature agree that inadequate training on opioid use for pain management is a major obstacle to opioid availability and is the root cause of lack of knowledge, inadequate pain assessments, opiophobia and under-prescribing among health professionals [14, 39].

### Healthcare system-related barriers

Judicial barriers such as over-regulation and legal restrictions of controlled medicines (e.g., opioids) are common in sub-Saharan African countries [20, 25, 28]. According to the 2022 report by the International Narcotic Control Board, 85.7% of global consumption of morphine for pain management is within a few high-income countries in North America and Europe, while 14.3% are used by low- and middle-income countries, including sub-Saharan African countries [40]. The non-clinical use of opioids has become a rapidly growing burden in sub-Saharan Africa due to the increasing utilisation of African trade routes for illegal opioid trafficking to Europe [41]. Thus, sub-Saharan Africa faces the double burden of devising strategies to curb the epidemic while improving access to opioids for pain relief in clinical settings.

Also, evidence suggest that pain management in cancer patients is hindered by logistical and procurement challenges surrounding morphine availability [21]. A significant challenge is the practice of projecting opioid need based on previous year's consumption [21]. A situation which might result in the underestimation of need if consumption levels were low in the previous year because of other barriers, such as high cost. Such underestimation may lead to opioid stockouts making it more difficult for cancer patients to access necessary opioids [20].

Furthermore, inadequate resource allocation, such as funding, significantly contributes to poor health service delivery in sub-Saharan Africa [42, 43]. Since direct OOP payment is the region's most used healthcare financing method, cancer patients are often saddled with the responsibility of paying for the prohibitive cost of opioid analgesics and other complementary drugs for opioid-related side effects, such as laxatives [25]. One study reported that OOP costs of medical care were the highest in the cancer patient group – with a mean cost of $207 compared with $55.4 for the general study population [29]. Other factors such as workforce shortages and urban-rural disparities in resource distribution within the health system could also limit the availability and accessibility to cancer pain management [21, 24, 25].

### Concept modelling

**Figure 3.**
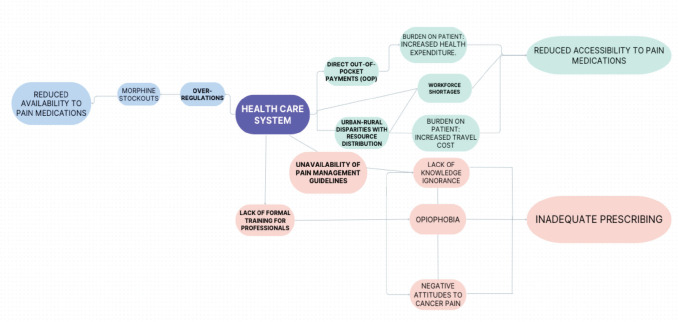
Concept map.

Following the analysis of existing evidence, several connections between the existing barriers were reported by the studies included in this review and depicted in Figure 3. Applying system-level thinking could help policymakers and future researchers focus on gaps in knowledge and evidence and, thus, implement strategies or conduct further analyses to address these barriers [44].

### Perspectives

The literature analyses uncovered that studies on cancer pain are closely linked to palliative care. Therefore, further research on the availability and quality of palliative care services in sub-Saharan Africa could enhance or differentiate the outcomes of this review.

### Limitations

The studies included in this review were conducted in specific locations, meaning their results may not be generalisable to other countries not represented in the data. Similarly, selection bias might have occurred at recruitment given that many of the papers were cross-sectional studies without randomisation.

Also, excluding publications from Francophone and Arabic countries due to the pre-specified language eligibility criterion could have limited the inclusion of potentially relevant studies from these countries. Only a database search was conducted; hence, grey literature and publications that could have been identified through other search methods such as, snowballing and citation searching, might have been omitted.

## Conclusion

The barriers to effectively managing cancer pain in sub-Saharan Africa cuts across several levels of the healthcare system, and evidence indicates connections between and within the significant domains identified in the literature. Health system-level barriers were found to be a significant contributor to inadequate cancer pain treatment. Some of the key findings of this review align with other systematic studies conducted within sub-Saharan Africa and on individuals of African descent elsewhere. Further high-quality research on system-level interventions to inadequate cancer pain management and related concepts, such as palliative care, is needed.

## Conflicts of interest

None declared.

## Funding

No funding was sought for this scoping review.

## Author contributions

VOM conceptualised the research topic under the supervisory guidance of PF, VOM and OAS independently conducted the screening of articles at both stages and quality assessment of the selected papers for the review. All disputes arising from both processes were resolved by PF. VOM wrote the various sections of the paper in fulfilment of her MSc programme. OAS, RK and PF critically reviewed the draft and made significant corrections, where appropriate. All authors gave the approval for the final draft to be published.

## Figures and Tables

**Table 1.** General study characteristics.

Study ID	Country	Setting	Study design	Study population	Sample size (n)
Abate *et al* [22]	Ethiopia	Hospitals (tertiary facility and specialised palliative care hospital), Hospice Ethiopia (non-governmental organisation (NGO))	Qualitative	Cancer patients Primary caregivers Healthcare providers Volunteers Nationwide palliative care advisors/advocates	25
Beck [24]	South Africa	Multiple professional settings	Qualitative	Health providers Academicians Government representatives NGO representatives Pharmaceutical representatives	33
Beck and Falkson [23]	South Africa	Phase 1: Oncology settings (private and public) Phase 2: Multiple sites (Phase I sites, public and private hospitals, hospices, local patients’ services division in four districts of Cancer Association of South Africa	Cross-sectional study	Cancer patients	426
Bell *et al* [33]	Ghana	Government tertiary facility	Cross-sectional study	Cervical cancer patients	100
Cleary *et al* [20]	25 African countries – Algeria, Botswana, Cote D'Ivoire, Egypt, Ethiopia, Ghana, Kenya, Liberia, Libya, Madagascar, Malawi, Mauritius, Morocco, Mozambique, Namibia, Nigeria, Rwanda, Sierra Leone, South Africa, Sudan, Swaziland, Tanzania, Tunisia, Uganda and Zimbabwe	Palliative and cancer care facilities	Cross-sectional study	Clinicians in the field of palliative/cancer care	**
Logie and Harding [25]	Uganda	Rural and urban hospice sites, pilot government district hospitals, the Ministry of Health and home-based care NGO	Mixed methods: Qualitative Cross-sectional study Quality audit	Phase 1: Clinicians; patients; key informants including senior clinical and governmental staff Phase 2: Direct observation of morphine entry Phase 3: Clinical care audit	42
Mwaka *et al* [26]	Uganda	District hospitals (missionary and public facilities)	Qualitative	Health professionals	15
Namisango *et al* [21]	Mozambique Swaziland Zimbabwe	**	Mixed methods: Cross-sectional study Qualitative Document review	Country representatives from government, law enforcement, health service and regulatory bodies	121
Ogboli-Nwasor et al [27]	Nigeria	Government tertiary facility	Cross-sectional study	Medical practitioners	82
Onsongo [28]	Kenya	Government tertiary facility	Qualitative	Private and oncology ward nurses	25
Reid *et al* [29]	Ethiopia	Oncology outpatient clinic Home Hospice Ethiopia	Mixed methods: Cross-sectional study Qualitative	Cancer patients	46
Tapera and Nyakabau [30]	Zimbabwe	Hospitals and hospice	Mixed methods: Cross-sectional studies Qualitative	Cervical cancer patients Men (patient’s partners) Healthy women Caregivers Health workers Stakeholders	296
Tuem *et al* [31]	Ethiopia	Oncology unit, tertiary hospital	Cross-sectional study	Cancer patients	91
Umar *et al* [32]	Kenya	Community-based	Cross-sectional study	Cancer patients	284

** Multiple reporters/settings per country, exact number/information not provided

**Table 2.** Summary of findings.

Study ID/year	Barriers			Critical appraisal rating (*)
	Patient-related	Professional-related	Healthcare system-related	
Abate *et al* 2023 [22]	Cultural beliefs and practices	-	Morphine supply shortages	*****
Beck 2000 [24]	Cultural beliefs and practices OOP health expenditure	Lack of interprofessional collaboration Poor doctor-patient relationship Lack of knowledge	Unavailable cancer pain management guidelines Limited health care resources Direct OOP payments	*****
Beck and Falkson 2001 [23]	-	Inadequate opioid prescription	-	*****
Bell *et al* 2022 [33]	Cultural beliefs and practices	Ignorance and negative attitude Lack of knowledge	-	*****
Cleary *et al* 2013 [20]	-	-	Legal restrictions and over-regulation of opioids	*****
Logie and Harding 2005 [25]	OOP health expenditure Travel cost	Opioid dispensing inconsistencies Opiophobia	Legal restrictions and over-regulation of opioids Direct OOP payments Limited health care resources Lack of staff training	***^a^
Mwaka *et al* 2013 [26]	-	-	Lack of established palliative care services Morphine supply shortages	*****
Namisango et al 2018 [21]	Travel cost	Opiophobia	Lack of established palliative care services Rural-urban imbalance with resource distribution Lack of staff training Limited health care resources Legal restrictions and over-regulation of opioids	****^b^
Ogboli-Nwasor *et al* 2013 [27]	-	Lack of knowledge Ignorance and negative attitude Opiophobia	Lack of staff training	****^c^
Onsongo 2020 [28]	Lack of knowledge Opiophobia	Poor doctor-patient relationship Lack of interprofessional collaboration Opiophobia Lack of motivation	Unavailable cancer pain. management guidelines Lack of staff training Human resource shortages Morphine supply shortages	*****
Reid *et al* 2018 [29]	OOP health expenditure	Inadequate opioid prescription	Direct OOP payments Medications stockouts/shortages	**^d^
Tapera and Nyakabau 2020 [30]	OOP health expenditure	-	Direct OOP payments Medications stockouts/shortages	****^e^
Tuem *et al* 2020 [31]	-	Inadequate opioid prescription	-	*****
Umar *et al* 2022 [32]	-	-	Travel restrictions during COVID-19 pandemic	***^f^

Methodology quality rating reporting [17]: 5 ***** or 100% quality criteria met; 4 **** or 80% quality criteria met; 3 *** or 60% quality criteria met; 2 ** or 40% quality criteria met; 1 * or 20% quality criteria met. Note: Mixed method studies were ranked based on the weakest component

a Can’t tell the relevance quantitative sample strategy and sample representative of the target population. No rationale for use of mixed method design. Quantitative component does not adhere to quality criteria

b Interpretation of result not substantiated by data. No rationale for use of mixed method design

c Can’t tell if the risk of nonresponse bias is low

d Can’t tell if qualitative data collection methods are adequate or findings are adequately derived and substantiated by data. Quantitative sample strategy not relevant to address research question. Also, can’t tell if the different research methods were adequately integrated and interpreted. Finally, both components do not adhere to quality criteria

e Unclear about the recruitment for survey – not clear if all the women coming to the clinic were surveyed. At risk of non-response bias due to recruitment strategy. Quantitative component does not adhere to quality criteria

f No information is provided on how participants were selected. Translation of tools without validation, social desirability bias especially for questionnaires administered by survey enumerators

## Appendix 1: Search strategy

**Table:**

Ovid MEDLINE(R) and Epub Ahead of Print, In-Process, In-Data-Review & Other Non-Indexed Citations, Daily and Versions <1946 to July 04, 2023>	Embase Classic+Embase <1947 to 2023 July 04>	# Web of Science Search Strategy (v0.1) Date Run: Tue Jul 04, 2023, 20:56:02 GMT+0100 (West Africa Standard Time)
1. exp Neoplasms/ 3852998 2. neoplasm*.tw. 157455 3. cancer.tw. 2113477 4. cancer surger*.tw. 16120 5. malignanc*.tw. 304670 6. tumo?r*.tw. 2015261 7. 1 or 2 or 3 or 4 or 5 or 6 4912758 8. Cancer Pain/ 2319 9. pain management*.tw. 31100 10. cancer pain.tw. 8857 11. (cancer pain adj3 management).tw. 1966 12. neoplasm pain.tw. 5 13. tumo?r pain.tw. 241 14. malignanc* pain.tw. 34 15. exp Pain Management 40852 16. ("pain relief" or "pain control").tw. 51651 17. 8 or 9 or 10 or 11 or 12 or 13 or 14 or 15 or 16 108566 18. (barrier? or challenge? or factor?).tw. 5156105 19. africa/ or "africa south of the sahara"/ or africa, central/ or cameroon/ or central african republic/ or chad/ or congo/ or "democratic republic of the congo"/ or equatorial guinea/ or gabon/ or "sao tome and principe"/ or africa, eastern/ or burundi/ or comoros/ or djibouti/ or eritrea/ or ethiopia/ or kenya/ or madagascar/ or rwanda/ or seychelles/ or somalia/ or south sudan/ or sudan/ or tanzania/ or uganda/ or africa, southern/ or angola/ or botswana/ or eswatini/ or lesotho/ or malawi/ or mozambique/ or namibia/ or south africa/ or zambia/ or zimbabwe/ or africa, western/ or benin/ or burkina faso/ or cabo verde/ or cote d'ivoire/ or gambia/ or ghana/ or guinea/ or guinea-bissau/ or liberia/ or mali/ or mauritania/ or niger/ or nigeria/ or senegal/ or sierra leone/ or togo/ 286414 20. 7 and 17 and 18 and 19 - **31**	1. exp Neoplasms/ 6021173 2. neoplasm*.tw.234798 3. cancer.tw. 3126038 4. cancer surger*.tw. 24712 5. malignanc*.tw.495142 6. tumo?r*.tw. 2917822 7. 1 or 2 or 3 or 4 or 5 or 7128299 8. Cancer Pain/ 23755 9. pain management*.tw.47455 10. cancer pain.tw. 15424 11. (cancer pain adj3 management).tw. 2757 12. neoplasm pain.tw. 4 13. tumo?r pain.tw. 393 14. malignanc* pain.tw. 59 15. exp Pain Management/213429 16. ("pain relief" or "pain control").tw. 80680 17. 8 or 9 or 10 or 11 or 12 or 13 or 14 or 15 or 16 278294 18. (barrier? or challenge? or factor?).tw. 6892810 19. africa/ or "africa south of the sahara"/ or africa, central/ or cameroon/ or central african republic/ or chad/ or congo/ or "democratic republic of the congo"/ or equatorial guinea/ or gabon/ or "sao tome and principe"/ or africa, eastern/ or burundi/ or comoros/ or djibouti/ or eritrea/ or ethiopia/ or kenya/ or madagascar/ or rwanda/ or seychelles/ or somalia/ or south sudan/ or sudan/ or tanzania/ or uganda/ or africa, southern/ or angola/ or botswana/ or eswatini/ or lesotho/ or malawi/ or mozambique/ or namibia/ or south africa/ or zambia/ or zimbabwe/ or africa, western/ or benin/ or burkina faso/ or cabo verde/ or cote d'ivoire/ or gambia/ or ghana/ or guinea/ or guinea-bissau/ or liberia/ or mali/ or mauritania/ or niger/ or nigeria/ or senegal/ or sierra leone/ or togo/ 386455 20. 7 and 17 and 18 and 19 - **115**	# Database: Web of Science Core Collection # Entitlements: - WOS.IC: 1993 to 2023 - WOS.CCR: 1985 to 2023 - WOS.SCI: 1900 to 2023 - WOS.AHCI: 1975 to 2023 - WOS.BHCI: 2005 to 2023 - WOS.BSCI: 2005 to 2023 - WOS.ESCI: 2015 to 2023 - WOS.ISTP: 1990 to 2023 - WOS.SSCI: 1900 to 2023 - WOS.ISSHP: 1990 to 2023 # Searches: 1: TS= (cancer OR tum?r* OR malignanc* OR "cancer and surger*" OR neoplasm*) Results: 4306256 2: TS= (cancer pain OR neoplasm pain OR cancer pain management OR malignanc* pain OR tumo?r pain OR pain relief OR pain control) Results: 279834 3: TS= (barrier? or challenge? or factor?) Results: 5527805 4: TS=(africa/ or "africa south of the sahara"/ or africa, central/ or cameroon/ or central african republic/ or chad/ or congo/ or "democratic republic of the congo"/ or equatorial guinea/ or gabon/ or "sao tome and principe"/ or africa, eastern/ or burundi/ or comoros/ or djibouti/ or eritrea/ or ethiopia/ or kenya/ or madagascar/ or rwanda/ or seychelles/ or somalia/ or south sudan/ or sudan/ or tanzania/ or uganda/ or africa, southern/ or angola/ or botswana/ or eswatini/ or lesotho/ or malawi/ or mozambique/ or namibia/ or south africa/ or zambia/ or zimbabwe/ or africa, western/ or benin/ or burkina faso/ or cabo verde/ or cote d'ivoire/ or gambia/ or ghana/ or guinea/ or guinea-bissau/ or liberia/ or mali/ or mauritania/ or niger/ or nigeria/ or senegal/ or sierra leone/ or togo/) Results: 967336 5: #1 AND #2 AND #3 AND #4 Results: **202**

## References

[1] World Health Organization (2018). WHO Guidelines for the Pharmacological and Radiotherapeutic Management of Cancer Pain in Adults and Adolescents.

[2] Bray F, Parkin DM, Gnangnon F (2022). Cancer in sub-Saharan Africa in 2020: a review of current estimates of the national burden, data gaps, and future needs. Lancet Oncol.

[3] Raja SN, Carr DB, Cohen M (2020). The revised IASP definition of pain: concepts, challenges, and compromises. Pain.

[4] Hadjiat Y, Perrot S (2022). Cancer pain management in French-speaking African countries: assessment of the current situation and research into factors limiting treatment and access to analgesic drugs. Front Public Health.

[5] Joseph AO, Salako O, Alabi A (2021). Cancer pain control in a Nigerian oncology clinic: treating the disease and not the patient. Pan Afr Med J.

[6] Soyannwo O (2009). Cancer pain – progress and ongoing issues in Africa. Pain Res Manag.

[7] World Health Organization (2021). MG 30.1 Chronic Cancer-Related Pain: International Classification of Diseases Eleventh Revision (ICD-11).

[8] Goudas LC, Bloch R, Gialeli-Goudas M (2005). The epidemiology of cancer pain. Cancer Investig.

[9] Stark L, Tofthagen C, Visovsky C (2012). The symptom experience of patients with cancer. J Hosp Palliat Nurs.

[10] Anekar AA, Hendrix JM, Cascella M (2023). WHO Analgesic Ladder.

[11] Cleary J (2000). Cancer pain management. Cancer Control.

[12] Koshy RC, Rhodes D, Devi S (1998). Cancer pain management in developing countries: a mosaic of complex issues resulting in inadequate analgesia. Support Care Cancer.

[13] Odonkor CA, Kim G, Erdek M (2017). Global cancer pain management: a systematic review comparing trials in Africa, Europe and North America. Pain Manag.

[14] Nchako E, Bussell S, Nesbeth C (2018). Barriers to the availability and accessibility of controlled medicines for chronic pain in Africa. Int Health.

[15] Tricco AC, Lillie E, Zarin W (2018). PRISMA extension for scoping reviews (PRISMA-ScR): checklist and explanation. Ann Intern Med.

[16] Bramer WM, Rethlefsen ML, Kleijnen J (2017). Optimal database combinations for literature searches in systematic reviews: a prospective exploratory study. Syst Rev.

[17] Hong QN, Fàbregues S, Bartlett G (2018). The mixed methods appraisal tool (MMAT) version 2018 for information professionals and researchers. Educ Inf.

[18] Page MJ, McKenzie JE, Bossuyt PM (2021). The PRISMA 2020 statement: an updated guideline for reporting systematic reviews. BMJ.

[19] Sun VC, Borneman T, Ferrell B (2007). Overcoming barriers to cancer pain management: an institutional change model. J Pain Symptom Manag.

[20] Cleary J, Powell RA, Munene G (2013). Formulary availability and regulatory barriers to accessibility of opioids for cancer pain in Africa: a report from the Global Opioid Policy Initiative (GOPI). Ann Oncol.

[21] Namisango E, Allsop MJ, Powell RA (2018). Investigation of the practices, legislation, supply chain, and regulation of opioids for clinical pain management in Southern Africa: a multi-sectoral, cross-national, mixed methods study. J Pain Symptom Manag.

[22] Abate Y, Solomon K, Azmera YM (2023). Barrier analysis for continuity of palliative care from health facility to household among adult cancer patients in Addis Ababa, Ethiopia. BMC Palliat Care.

[23] Beck SL, Falkson G (2001). Prevalence and management of cancer pain in South Africa. Pain.

[24] Beck SL (2000). An ethnographic study of factors influencing cancer pain management in South Africa. Cancer Nurs.

[25] Logie DE, Harding R (2005). An evaluation of a morphine public health programme for cancer and AIDS pain relief in Sub-Saharan Africa. BMC Public Health.

[26] Mwaka AD, Wabinga HR, Mayanja-Kizza H (2013). Mind the gaps: a qualitative study of perceptions of healthcare professionals on challenges and proposed remedies for cervical cancer help-seeking in post conflict northern Uganda. BMC Fam Pract.

[27] Ogboli-Nwasor E, Makama JG, Yusufu LMD (2013). Evaluation of knowledge of cancer pain management among medical practitioners in a low-resource setting. J Pain Res.

[28] Onsongo LN (2020). Barriers to cancer pain management among nurses in Kenya: a focused ethnography. Pain Manag Nurs.

[29] Reid EA, Gudina EK, Ayers N (2018). Caring for life-limiting illness in Ethiopia: a mixed-methods assessment of outpatient palliative care needs. J Palliat Med.

[30] Tapera O, Nyakabau AM (2020). Limited knowledge and access to palliative care among women with cervical cancer: an opportunity for integrating oncology and palliative care in Zimbabwe. BMC Palliat Care.

[31] Tuem KB, Gebremeskel L, Hiluf K (2020). Adequacy of cancer-related pain treatments and factors affecting proper management in Ayder Comprehensive Specialized Hospital, Mekelle, Ethiopia. J Oncol.

[32] Umar S, Chybisov A, McComb K (2022). COVID-19 and access to cancer care in Kenya: patient perspective. Int J Cancer.

[33] Bell SG, AppiahKubi A, Konney TO (2022). Barriers to adequate pain control among women with cervical cancer: exploring unmet pain control needs in Ghana. AJOG Glob Rep.

[34] Mitchell R, Rose P, Asare S (2020). Education research in Sub-Saharan Africa: quality, visibility, and agendas. Comp Educ Rev.

[35] Nortjé N, Albertyn R (2015). The cultural language of pain: a South African study. S Afr Fam Pract.

[36] Kwok W, Bhuvanakrishna T (2014). The relationship between ethnicity and the pain experience of cancer patients: a systematic review. Indian J Palliat Care.

[37] Ratshikana-Moloko M, Ayeni O, Tsitsi JM (2020). Spiritual care, pain reduction, and preferred place of death among advanced cancer patients in Soweto, South Africa. J Pain Symptom Manag.

[38] Lombe DC, Mwamba M, Msadabwe S (2023). Delays in seeking, reaching and access to quality cancer care in sub-Saharan Africa: a systematic review. BMJ Open.

[39] International Narcotics Control Board (2023). Supplement to the Annual Report of the Board for 2022 on the Availability of Internationally Controlled Substances. No Patient Left Behind: Progress in Ensuring Adequate Access to Internationally Controlled Substances for Medical and Scientific Purposes.

[40] International Narcotics Control Board (2022). Report of the International Narcotics Control Board for 2022.

[41] Kurth AE, Cherutich P, Conover R (2018). The opioid epidemic in Africa and its impact. Curr Addict Rep.

[42] Oleribe OO, Momoh J, Uzochukwu BS (2019). Identifying key challenges facing healthcare systems in Africa and potential solutions. Int J Gen Med.

[43] Azevedo MJ (2017). The State of Health System(s) in Africa: Challenges and Opportunities. Historical Perspectives on the State of Health and Health Systems in Africa.

[44] Peters DH (2014). The application of systems thinking in health: why use systems thinking?. Health Res Policy Sys.

